# Uncorrelated inputs enhance signal representation in the inhibitory striatum network

**DOI:** 10.1186/1471-2202-12-S1-P182

**Published:** 2011-07-18

**Authors:** Man Yi Yim, Ad Aertsen, Arvind Kumar

**Affiliations:** 1 Bernstein Center Freiburg, University of Freiburg, Freiburg, 79104, Germany; 2 Neurobiology & Biophysics, Faculty of Biology, University of Freiburg, Freiburg, 79104, Germany

## 

Medium spiny neurons (MSNs) are the principal neurons in the striatum. The recurrent connections among them mediate weak feedback inhibition, whereas fast-spiking interneurons (FSIs) project extensively to the MSNs and provide strong feedforward inhibition. The striatum is innervated by massive excitatory afferents from various regions of the neocortex via the corticostriatal projection neurons. Interestingly, despite their strong convergence, corticostriatal projections are structured in such a way that neighboring MSNs in the striatum are not likely to share their presynaptic inputs [1].

To understand the functional consequences of such a corticostriatal connectivity structure we studied the representation of cortical inputs in a spiking neural network model of the striatum. Activation of a fraction of MSNs resulted in a corresponding decrease in the spiking of unstimulated neurons, due to the overall inhibitory nature of the striatum network. This was similar to the neuronal activity recorded in behaving rats [2]. Further, we found that correlations in the cortical inputs strongly influenced the transfer function of the striatum. Weak pairwise correlation within the input pool of individual striatal neurons enhanced the representation by increasing the signal-to-noise ratio (activity of stimulated vs. unstimulated neurons) in the striatum. By contrast, correlation among the input pools of different neurons weakened the representation, because the resulting synchronized inhibition was less efficient to silence the unwanted background activity. Interestingly, the structure of corticostriatal projections provides the conditions that minimize the correlation among the input pools of different MSNs.

MSNs can display short epochs of rapid firing, which may change their overall response to cortical inputs. Our simulation results suggest that the bursting activity of the MSNs did not affect the correlation dependence of the signal representation. Rather, it modulated the representation in a qualitative manner. In addition to the correlation of the excitatory inputs, synchrony within the inhibitory striatal network also influenced the signal representation. Specifically, we show that correlated feedforward inhibition mediated by FSIs, which might arise due to gap junction couplings, impaired the signal representation in the striatum. To date, experimental [3] and computational [4] studies suggest that feedforward inhibition is not correlated. According to our model, this supports an optimal representation of cortical inputs in the striatal network.

In summary, we showed that uncorrelated excitation and uncorrelated inhibition in the striatum support optimal signal representation, which, based on the anatomical and physiological findings, may be utilized by the striatum to enhance the encoding of cortical information for the execution of different cognitive functions.
